# Generative Adversarial Network for Synthesizing Multivariate Time-Series Data in Electric Vehicle Driving Scenarios

**DOI:** 10.3390/s25030749

**Published:** 2025-01-26

**Authors:** Shyr-Long Jeng

**Affiliations:** Department of Mechanical Engineering, Lunghwa University of Science and Technology, Taoyuan City 333326, Taiwan; aetsl@gm.lhu.edu.tw; Tel.: +886-2-8209-3211 (ext. 5113)

**Keywords:** conditional generative adversarial networks, principal component analysis (PCA), SOC estimation, time-series synthesis

## Abstract

This paper presents a time-series point-to-point generative adversarial network (TS-p2pGAN) for synthesizing realistic electric vehicle (EV) driving data. The model accurately generates four critical operational parameters—battery state of charge (SOC), battery voltage, mechanical acceleration, and vehicle torque—as multivariate time-series data. Evaluation on 70 real-world driving trips from an open battery dataset reveals the model’s exceptional accuracy in estimating SOC values, particularly under complex stop-and-restart scenarios and across diverse initial SOC levels. The model delivers high accuracy, with root mean square error (RMSE), mean absolute error (MAE), and dynamic time warping (DTW) consistently below 3%, 1.5%, and 2.0%, respectively. Qualitative analysis using principal component analysis (PCA) and t-distributed stochastic neighbor embedding (t-SNE) demonstrates the model’s ability to preserve both feature distributions and temporal dynamics of the original data. This data augmentation framework offers significant potential for advancing EV technology, digital energy management of lithium-ion batteries (LIBs), and autonomous vehicle comfort system development.

## 1. Introduction

The escalating global energy demand and environmental challenges posed by climate change have intensified the search for clean energy solutions and efficient storage technologies. Lithium-ion batteries (LIBs), with their broad operating temperature range, extended cycle life, and high energy density, have emerged as a cornerstone of the green energy transition. While these batteries power diverse applications from consumer electronics to spacecraft, their role in electric vehicles (EVs) is particularly crucial. Industry and academic researchers have made significant strides in enhancing EV safety, performance, and range. The continuous advancement of EV technologies—including battery systems, autonomous capabilities, and other innovations—underscores their fundamental role in achieving a sustainable future.

State of charge (SOC) estimation is crucial for battery management systems (BMSs), particularly in EV applications. Various estimation methods [1,2,3] have emerged, ranging from simple direct measurements to sophisticated algorithmic approaches. Direct measurement monitors voltage or current, offering simplicity but limited accuracy due to non-linear voltage−SOC relationships and environmental influences. Coulomb counting integrates current overtime for real-time estimation but requires accurate initial SOC to prevent drift. The open-circuit voltage (OCV) method correlates resting voltage with SOC, providing good accuracy but requiring extended rest periods and showing sensitivity to temperature and aging effects.

Advanced estimation techniques have evolved to address these limitations. Kalman filtering approaches [4], including Extended Kalman Filter (EKF) and Unscented Kalman Filter (UKF), combine measurement data with battery models for dynamic estimation, offering noise resilience but requiring precise modeling and substantial computation. Observer-based methods, including Luenberger and sliding mode approaches, provide robust performance under dynamic conditions while depending on accurate system models. Electrochemical modeling delivers unmatched accuracy through fundamental equation solving but involves complex implementation and heavy computational load.

Hybrid methods have emerged as a promising solution, combining multiple approaches such as Coulomb counting with Kalman filtering to optimize accuracy, robustness, and computational efficiency. The selection of an appropriate SOC estimation method ultimately depends on application-specific requirements. While basic applications may find simpler methods sufficient, high-performance EVs typically require advanced techniques to ensure reliable operation across diverse conditions.

Recent advances in machine learning have demonstrated significant potential for improving SOC estimation in LIBs without explicit modeling, though these approaches demand substantial computational resources despite their high accuracy with sufficient training data. The complexity and variability of real-world LIBs and EV time-series data present significant challenges for both comprehensive dataset collection and model reliability. Sarda et al. [5] highlighted critical challenges in their comprehensive review, particularly emphasizing non-linear battery behavior and evolving battery management system (BMS) requirements, while Obuli et al. [6] identified Gaussian Process Regression as particularly effective despite challenges with variable driving conditions. Khan et al. [7] advanced the field through innovations in deep neural networks, addressing battery behavior in dynamic environments and proposing novel approaches to architectural design and hyperparameter optimization. Selvaraj et al. [8] further enhanced this progress by implementing Bayesian methods for hyperparameter tuning, demonstrating robust SOC predictions across diverse operational conditions, including varying vehicle velocities and environmental factors.

To address real-world data limitations stemming from the high cost and time demands of data collection, researchers have increasingly turned to data augmentation techniques as a cost-effective alternative to extensive physical testing. Iglesias et al. [9] demonstrated the effectiveness of various augmentation methods, including adversarial and automatic approaches, in generating synthetic data that better represent time-series patterns, building upon Wen et al.’s [10] foundational taxonomy of time-domain, frequency-domain, and advanced decomposition-based methods. Lee et al. [11] introduced Temporal Adversarial Data Augmentation (TADA), leveraging time warping to better simulate real-world variations, while Victor et al. [12] provided a comprehensive framework for data augmentation across multiple data types, demonstrating its effectiveness in various machine learning tasks through detailed case studies. Domain-Adaptive Designable Data Augmentation (DADDA) [13] integrates inverse generation with domain adaptation to facilitate rapid design solutions for advanced EV systems. However, accurately modeling dynamic responses across diverse driving conditions remains a significant challenge.

Generative Adversarial Networks (GANs) have revolutionized data synthesis, enabling the creation of realistic images, audio, videos, and more [14]. These models are widely applied in fields such as cybersecurity for intrusion and anomaly detection [15], medical imaging for data augmentation and disease diagnosis [16], and healthcare for synthesizing patient time-series data [17]. GANs excel at generating synthetic data that closely resemble real-world distributions, providing scalable solutions when collecting real data is costly, hazardous, or ethically challenging [18,19]. The framework consists of two neural networks: a generator that produces synthetic data by mapping random noise to the target data distribution and a discriminator that evaluates whether the input data are real or synthetic. These networks compete in a minimax game where the generator aims to fool the discriminator, and the discriminator seeks to distinguish real data from synthetic. This adversarial training allows GANs to model complex data distributions without explicit density estimation. Time-series GANs have demonstrated significant utility in improving energy storage predictions by integrating physical and digital systems [20]. Advanced architectures such as Progressive GANs and StyleGANs have enabled high-resolution image generation and fine-grained control over synthetic outputs [21]. Despite challenges like training instability and mode collapse [22], GANs continue to evolve, incorporating techniques like reinforcement learning and convolutional neural networks to enhance their capabilities [23]. Their ability to generate high-quality synthetic data has made GANs a cornerstone of unsupervised learning, driving innovations across various domains.

Researchers have made significant strides in generating battery time-series data to improve SOC estimation for LIBs. Wong et al. [24] combined a generative GAN (gGAN) with an SOC estimator, generating realistic battery features like voltage, current, and temperature essential for accurate SOC calculations. However, ensuring the synthetic data accurately reflects real-world conditions remains challenging, as discrepancies can impact model performance. The TS-DCGAN model [25] generates synthetic battery data to enhance SOC estimator training. While this approach addresses the scarcity of high-quality datasets, it requires significant computational resources and expertise for integration into existing frameworks. Similarly, TimeGAN [26] generates synthetic time-series data while maintaining realistic temporal dependencies and feature correlations, though generating long sequences with complex temporal relationships remains difficult.

Recent developments in GAN architecture have focused on addressing specific challenges in battery system applications. Wasserstein GAN (WGAN) [27] adapts to the sequential nature of SOC data, preserving long-term dependencies and improving the accuracy of SOC estimation models. However, synthetic data may still struggle to replicate rare or edge cases, limiting its effectiveness under variable operating conditions. Soo et al. [28] introduced a modified TimeGAN that generates virtual battery datasets to enhance model training with limited real data, though such synthetic data may not fully represent real-world scenarios. ITF-GAN [29] combines a deep autoencoder with GANs to generate synthetic data, while Zhang et al. [30] developed the TimesNet model, a deep learning framework enhanced with Gaussian noise-based data augmentation to simulate diverse operating conditions.

Accurate SOC estimation in LIBs is crucial for the safe and efficient operation of EVs. While laboratory settings offer controlled conditions for SOC characterization, real-world driving presents significant challenges. Variable initial SOCs, temperature fluctuations, unpredictable load variations, and diverse driving patterns distort voltage signals, rendering conventional SOC estimation methods unreliable. Existing studies often simplify scenarios with constant loads or predetermined SOC levels, which do not generalize well to the complexities of real-world driving. This discrepancy between controlled experiments and real-world conditions creates a critical need for robust SOC estimation methods capable of handling diverse and noisy data. The primary objective of this study is to develop a novel data augmentation technique using synthetic data to improve the accuracy and robustness of SOC estimation models in real-world EV applications. Generating synthetic data mitigates the limitations associated with relying exclusively on real-world datasets, which are often constrained in terms of size, diversity, and their ability to represent all possible driving conditions. Training models on a combination of real and synthetic data enhance their generalization capabilities for unseen real-world scenarios, thereby improving the accuracy of SOC estimation under challenging conditions.

GANs have shown promise in generating synthetic data across various domains, including image synthesis and time-series generation. Building upon the Pix2Pix architecture [31], which demonstrated successful image-to-image translation, subsequent works like HR-Pix2Pix [32] and Ambient-Pix2PixGAN [33] extended its capabilities to high-resolution images and medical imaging with noisy inputs, respectively. However, challenges related to training stability and generalization to real-world data persisted [34]. These studies highlight the potential of GANs for data augmentation but also emphasize the importance of careful model design and evaluation to ensure the quality and utility of the generated data. To address the specific challenges of time-series data in EV SOC estimation, TS-p2pGAN, a novel GAN-based framework, was developed for augmenting multivariate time-series data.

This model synthesizes dynamic data representing real-world driving scenarios, enabling more effective training of deep learning-based SOC estimation models. TS-p2pGAN integrates environmental, vehicle, battery, and heating system variables, concatenating them with time-series features to generate synthetic SOC and motion data. It ensures robust temporal dependencies among variables and accommodates varying sequence lengths, offering efficient representations of complex time-series data. By training on historical time-series data, TS-p2pGAN captures temporal patterns and generates plausible future trajectories, enhancing dataset diversity and machine learning model performance, especially when real-world data collection is challenging. This capability makes TS-p2pGAN suitable for a wide range of time-series analysis and prediction tasks.

The key contributions of this study are as follows:

- Data augmentation framework for EV driving scenarios: A GAN-based framework for synthesizing multivariate time-series data specifically for EV driving scenarios is presented. This framework addresses the limited availability of real-world data by enabling the generation of larger and more diverse datasets suitable for training and evaluating SOC estimation models.
- TS-p2pGAN model architecture: The TS-p2pGAN model, incorporating an integrated transformation network and a multiscale discriminator, is designed to handle high-dimensional, extended time sequences. This architecture aims to capture complex temporal dependencies within the data and generate synthetic time series that preserve these dependencies.
- Evaluation protocol using quantitative and qualitative metrics: An evaluation protocol employing both quantitative and qualitative metrics is implemented to assess the quality and characteristics of the generated synthetic data. This protocol provides insights into the model’s performance and facilitates further development.
- Validation with real-world driving data: The model’s performance is evaluated using data from 70 real-world driving trips, demonstrating its ability to generalize to real-world conditions and its potential for practical application in EV SOC estimation.

The remainder of this paper is organized as follows: Section 2 introduces the dataset and provides details of the TS-p2pGAN architecture and training methodology. Section 3 covers the experimental setup, evaluation metrics, and both quantitative and qualitative results. Finally, Section 4 concludes with a summary of the findings and potential directions for future research.

## 2. Materials and Methods

### 2.1. Dataset

The study utilizes a publicly available dataset from IEEE DataPort [35] containing comprehensive measurements of high-voltage battery performance and vehicle behavior for a BMW i3 (60 Ah) EV. The BMW i3 (60 Ah) EV [36] produced between 2013 and 2017 is a compact electric vehicle known for its innovative design and efficient performance. An overview of the key performance, battery, dimension, and charging parameters for the BMW i3 (60 Ah) is summarized in Table 1.

The dataset comprises 70 trips conducted during summer (Group A) and winter (Group B), capturing both powertrain and heating circuit data under real-world driving conditions. Group A includes 32 trips, labeled trip A01 to A32, while Group B comprises 38 trips, labeled trip B01 to B38. While the battery loads showed significant fluctuations throughout operation, auxiliary system consumption—particularly from heating and air-conditioning systems—maintained stable patterns but substantially impacted the vehicle range.

While the original dataset includes 30 variables per trip, this study focuses on key parameters essential for the TS-p2pGAN model implementation:

- Vehicle dynamics: speed, altitude, throttle position, motor torque, and longitudinal acceleration;
- Battery metrics: power battery voltage, current, temperature, actual SOC, and displayed SOC;
- Climate control: heater wattage demand, air conditioner power consumption, heater voltage, and current;
- Environmental conditions: ambient temperature and related parameters.

**Figure 1:**
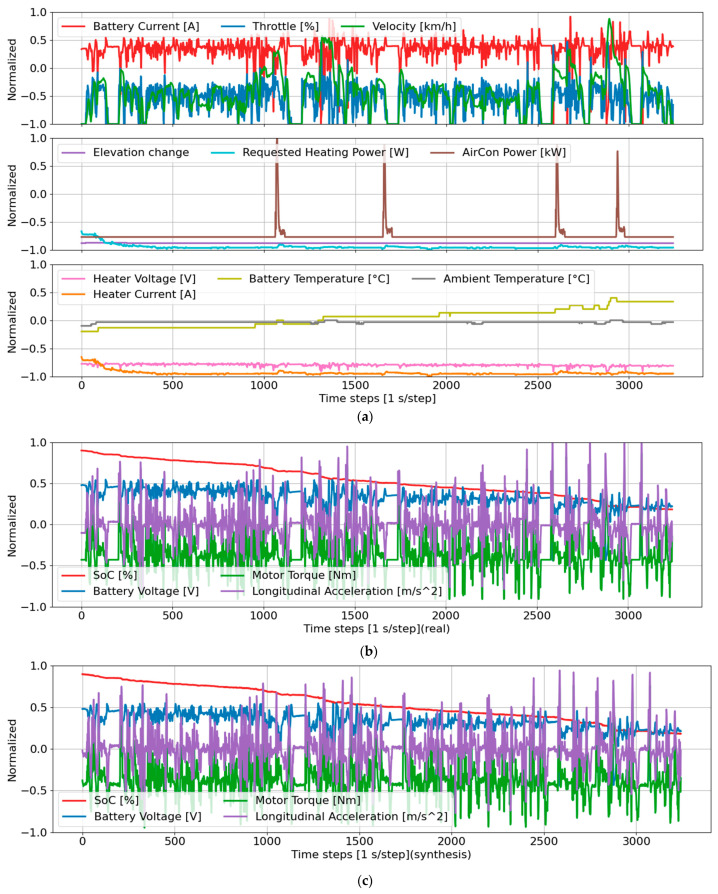
Multivariate time series illustrating the input and output data of trip B01 for the TS-p2pGAN model: (**a**) Time-series input data comprising ten features; (**b**) ground truth (real) time series comprising four features; (**c**) synthetic time series comprising four features.

The data were used to train the TS-p2pGAN model, as illustrated in Figure 1. The generator’s inputs included ten features, shown in Figure 1a. Figure 1b,c present the real signals and the synthetic signals generated by the model, respectively. All parameters were normalized to the range of [−1, 1].

### 2.2. Time-Series Synthesis with the pix2pix GAN

While the pix2pix GAN [31] was originally designed for image-to-image translation tasks, its framework has been extended to accommodate multivariate one-dimensional (1D) time-series transformations. Unlike conventional GANs that generate data solely from random noise vectors, the pix2pix GAN employs a conditional framework where the network learns direct mappings between input and output data pairs. This conditioning approach enables more realistic and controlled data generation, making it particularly suitable for time-series translation tasks.

**Figure 2:**
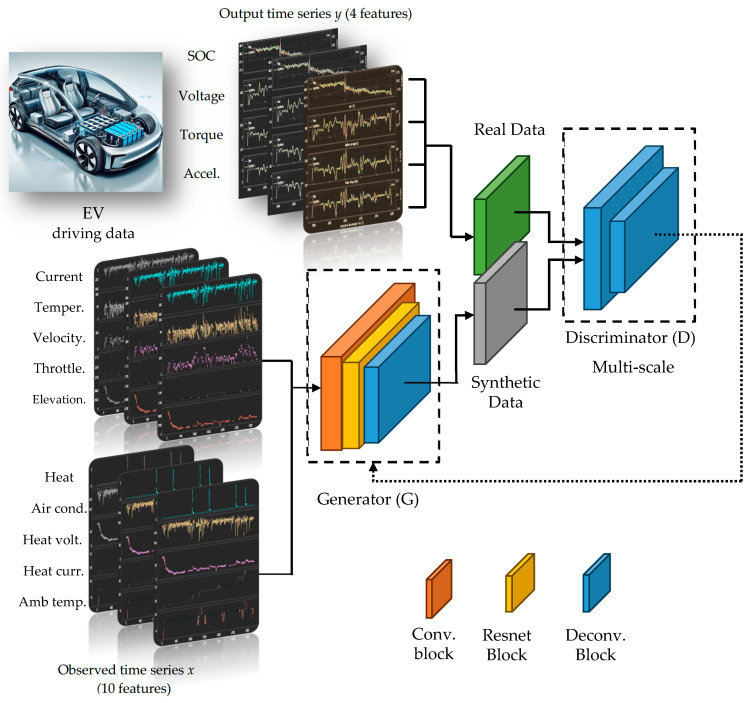
Framework of TS-p2pGAN.

The proposed TS-p2pGAN model, illustrated in Figure 2, functions as a two-player adversarial game between a generator (*G*) and a discriminator (*D*). The core objective is to learn a mapping function that transforms an observed time series *x* and a random noise vector *z* into a corresponding output time series *y*, expressed mathematically as *G*: {*x*, *z*} → *y*. The generator accepts two inputs: random noise sampled from a Gaussian distribution and conditioning information from time-series data. Through training, the generator learns to approximate the underlying probability distribution of real data and produces synthetic data samples guided by the conditioning constraints. Meanwhile, the discriminator evaluates both real and generated data samples, incorporating conditioning information in its assessment as it learns to distinguish between authentic and synthetic time series.

The conditioning mechanism provides crucial constraints for the generation process, allowing specific characteristics to be targeted in the output time series. This approach ensures that the generated data maintain meaningful relationships with the input time series while exhibiting natural variations and realistic features. By leveraging this conditional framework, TS-p2pGAN can effectively translate between different time series domains while preserving the essential characteristics and temporal dependencies of the data.

The framework is a supervised learning algorithm, with its training dataset comprising pairs of time series {(*x*, *y*)}, where *x* is a conditioning time series and *y* is a corresponding real time series. The objective function of TS-p2pGAN [32] can be formulated as follows:

(1) $$L_{TS-p2pGAN}\left(G,D\right)=E_{x,y}\left[logD(x,y)\right]+E_{x,y}\left[log(1-D(x,G(x,z))\right]$$

where 

x is the real data from the dataset;

y is the synthetic data;

z is the latent space (noise) input to the generator G(x,z);

D(x,y) is the discriminator’s probability that x is real and y is the synthetic data;

G(x,z) is the generated data from the generator G(x,z) given the noise z and real x;

$D(x,G\left(x,z\right))$ is the discriminator’s probability that the generated data are real.

The generator *G* seeks to minimize this objective, as given in Equation (1), while the discriminator D endeavors to maximize it, as expressed in the following Equation:

(2) $$G^{*}=arg\;\underset{G}{\min}\underset{D}{\max}L_{TS-p2pGAN}\left(G,D\right)$$

The generator’s objective is to not only deceive the discriminator but also closely approximate the ground truth output in terms of the *L*_1_ distance:

(3) $$L_{L1}\left(G\right)=E_{x,y,z}\left[{\left\|y-G(x,z)\right\|}_{1}\right]$$

According to Equations (2) and (3), the final objective is thus expressed as follows:

(4) $$G^{*}=arg\;\underset{G}{\min}\underset{D}{\max}L_{TS-p2pGAN}\left(G,D\right)+\lambda L_{L1}\left(G\right)$$

The TS-p2pGAN objective function in Equation (4) is a minimax game, where the generator tries to generate data that are indistinguishable from real data, and the discriminator tries to distinguish between real and fake data. Both the generator and the discriminator are trained simultaneously to improve each other’s performance.

Noise (*z*) may be incorporated in accordance with the specific application and desired characteristics of the generated data; noise can introduce randomness and enhance sample diversity. However, noise is unnecessary for TS-p2pGAN because it receives both input data and conditioning information to generate output data and can learn the mapping between *x* and *y* without noise [32]. In the model, noise is introduced exclusively through dropout, applied to multiple generator layers during both the training and testing phases. Despite the inclusion of dropout noise, only minimal stochasticity was observed.

#### 2.2.1. Architecture of the Transformation Net Model in the Generator Framework

The 1D generator in the TS-p2pGAN architecture builds upon the foundational image translation framework [31,32], adapting it for time-series applications. This generator performs domain-to-domain translation of temporal data sequences through three primary components: a convolutional front-end encoder (*GF*), a sequence of residual blocks (*GR*) for feature processing, and a transposed convolutional back-end decoder (*GB*). The detailed specifications of each component are presented in Table 2.

The front-end encoder begins with an input layer that processes batches of 256 samples, each containing 10 features. A 1D reflective padding layer (Reflect_1; padding size: 3) preserves boundary information for subsequent convolutions. The initial convolutional layer (Conv_1) employs a kernel size of 7 and a stride of 1 to generate 64 features while maintaining the 256-dimensional structure. Following this, Conv_2 through Conv_5 perform progressive downsampling using kernels with a size of 3, stride 2, and padding 1, reducing dimensions from 128 to 16 while enriching feature representations. Each convolution incorporates BatchNorm1d for training stability and ReLU activation for nonlinear feature transformation.

The intermediate stage consists of nine residual blocks (ResnetBlock_1 to ResnetBlock_9) that preserve feature dimensionality while preventing vanishing gradients. Each block contains two Conv1d layers (kernel size: 3; stride: 1; padding: 1) with BatchNorm1d and ReLU activation functions. A skip connection bridges the input and output of each block, facilitating gradient flow and enabling efficient residual learning instead of direct input−output mapping.

The back-end decoder employs four transposed convolution layers (ConvTran_1 to ConvTran_4) to restore spatial resolution. Each layer operates with a kernel size of 3, a stride of 2, and a padding of 1, progressively doubling spatial dimensions while preserving detailed features. BatchNorm1d and ReLU activations follow each transposed convolution. The architecture concludes with a final convolution layer (Conv_6; kernel size: 7; no padding) that produces 4 features at 256 dimensions. A hyperbolic tangent activation function (Tanh_1) normalizes the output to the range [−1, 1], completing the time-series translation process.

#### 2.2.2. Multiscale Discriminators

The TS-p2pGAN framework incorporates a multiscale discriminator architecture to evaluate the authenticity of generated time-series data across different temporal scales. This architecture employs two parallel discriminators (*D*1 and *D*2) that process input data at different resolutions while sharing the same structural design. The system creates a two-scale time-series pyramid by downsampling the input data by a factor of 2, enabling the evaluation of both fine-grained details and broader temporal patterns. The coarse-scale discriminator operates with an expanded receptive field to ensure global consistency, while the fine-scale discriminator focuses on local detail refinement. This dual-scale approach provides comprehensive feedback to the generator for producing realistic time-series outputs.

The detailed architecture of both discriminator branches is specified in Table 3. “2. The initial three layers employ a kernel size of 4, a stride of 2, and a padding of 2, with the first layer expanding the feature dimension to 64 while reducing temporal dimensions to 129. The coarse discriminator (*D*2) begins with an average pooling operation (Pool1D_1; kernel size: 3; stride: 2; no padding) that downsamples the input to 128 temporal dimensions while preserving the 14 feature channels. This pooled input then passes through a parallel set of convolutional layers (Conv_6 to Conv_10) with specifications matching those of the fine discriminator branch, adjusted for the reduced input dimensions.

Both discriminator branches utilize Leaky ReLU activation (Conv1dR) after each convolutional layer, with BatchNorm1d normalization added in specific layers (Conv1dN + R). The final layer in each branch implements a sigmoid activation function, producing probability outputs suitable for cross-entropy loss calculation during training.

## 3. Experiments and Results

### 3.1. Data Preprocessing

The dataset comprises 70 trips from both Group A and Group B, with measurements recorded at 100 ms intervals. A simple 1 s moving average filter (ten-point window) [37] was applied to reduce noise and emphasize underlying trends, similar to the noise reduction techniques employed in microgrid applications with fluctuating renewable energy sources. The smoothed data were then segmented into overlapping sequences of 256 consecutive samples with a stride of 1, ensuring comprehensive temporal coverage while preserving sequential relationships. All input features were normalized to the range [−1, 1], resulting in a total of 87,536 segmented sequences for analysis.

The preprocessed sequences were used to train the TS-p2pGAN model. The generator, depicted in Figure 1a, accepts ten input features and produces four target signals: SOC, battery voltage, motor torque, and longitudinal acceleration (Figure 1b). Visual analysis of the synthetic data generated by the model (Figure 1c) reveals a remarkable alignment with the original signals, demonstrating the TS-p2pGAN’s capability to generate highly realistic and accurate time-series representations.

All implementations were performed using Python 3.11.6 and the PyTorch 2.1 library. Experiments were conducted on a system equipped with an Intel Core i7-10700K CPU and an NVIDIA GeForce RTX 4090 GPU with 24 GB VRAM. The Adam optimizer was employed for loss minimization, with the discriminator and generator initialized at a learning rate of 0.0006. The learning rate followed a gradual decay schedule, tapering to 0 during the final 200 epochs of the total 800-epoch training process. A batch size of 256 was used, with the dataset fully reshuffled between epochs to ensure training stability and mitigate overfitting in both networks.

### 3.2. Quantitative and Qualitative Performance Metrics

The TS-p2pGAN model’s performance was evaluated through both quantitative and qualitative metrics to assess the fidelity of synthetic time-series data. Three quantitative metrics—root mean square error (RMSE), mean absolute error (MAE), and dynamic time warping (DTW) [38]—were utilized to measure the deviation between real and synthetic data.

RMSE quantifies the average magnitude of prediction errors by calculating the square root of the mean squared differences:

(5) $$RMSE=\sqrt{\frac{1}{n}\sum_{i=1}^{n}{\left(y_{i}-{\hat{y}}_{i}\right)}^{2}}$$

where *n* represents the number of data points, *y_i_* demotes the actual value, and ${\hat{y}}_{i}$ represents the predicted value.

MAE measures the average absolute difference between predicted and actual values:

(6) $$MAE=\frac{1}{n}\sum_{i=1}^{n}\left|y_{i}-{\hat{y}}_{i}\right|$$

DTW is a powerful algorithm designed to measure the similarity between two temporal sequences by finding their optimal alignment. Unlike standard distance measures that compare sequences at fixed time points, DTW allows for flexible stretching or compressing of sequences non-linearly, enabling it to find the best match. This makes DTW particularly valuable for analyzing time-series data. The DTW algorithm [38] minimizes the total distance between corresponding points of the sequences under certain constraints. The recurrence relation for DTW is given by:

(7) $$D\left(i,j\right)=\left\|y_{i}-{\hat{y}}_{j}\right\|+min\left\{D\left(i-1,j\right),D\left(i,j-1\right),\left(i-1,j-1\right)\right\}$$

where $D\left(i,j\right)$ represents the cumulative distance up to the *i*-th and *j*-th points of the sequences, $y_{i}$ and ${\hat{y}}_{j}$ are the respective elements, and $\left\|\cdot \right\|$ denotes the distance. By using this recurrence relation, DTW identifies the optimal alignment path that minimizes the cumulative distance between the two sequences. These metrics provide objective measures of how accurately the model reproduces the statistical properties and temporal dependencies of the original dataset.

For qualitative assessment, the generated samples were visualized using two dimensionality reduction techniques: t-distributed stochastic neighbor embedding (t-SNE) [39] and principal component analysis (PCA) [40]. While PCA identifies linear projections that maximize explained variance, t-SNE specializes in preserving both local and global topological relationships when mapping high-dimensional data to lower dimensions. The visualizations from both techniques revealed that the synthetic data distributions closely aligned with those of the real observations, suggesting successful replication of the underlying data.

### 3.3. Real and Synthetic Data

**Figure 3:**
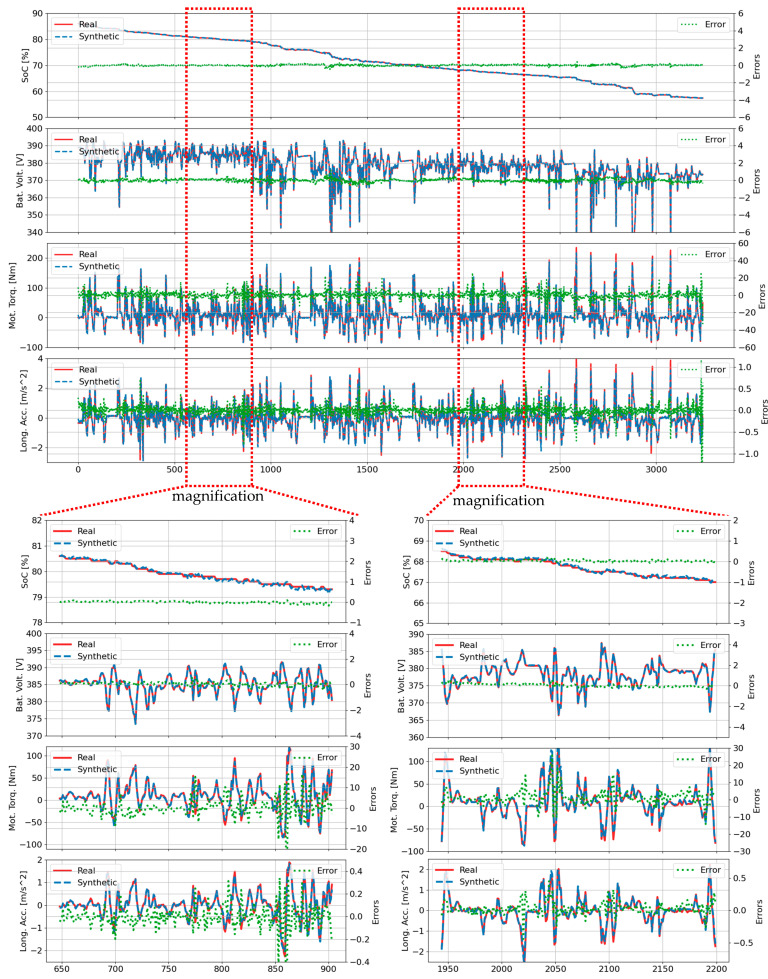
Comparison of real values and synthetic data generated by TS-p2pGAN for the B01 trip.

A detailed comparison between synthetic and actual waveforms for four key parameters (SOC, battery voltage, motor torque, and longitudinal acceleration) during trip B01 is presented in Figure 3. The green line indicates the deviation between synthetic and actual values, with detailed views highlighting model performance during rapid state changes across two specific time intervals: (648, 904) and (1942, 2198) in the lower left and right plots. The TS-p2pGAN model demonstrated exceptional reliability, particularly in SOC prediction, maintaining deviations within a narrow range of (−0.7, 0.1). While the synthetic waveforms for motor torque and longitudinal acceleration closely tracked actual patterns, minor magnitude discrepancies were observed.

**Figure 4:**
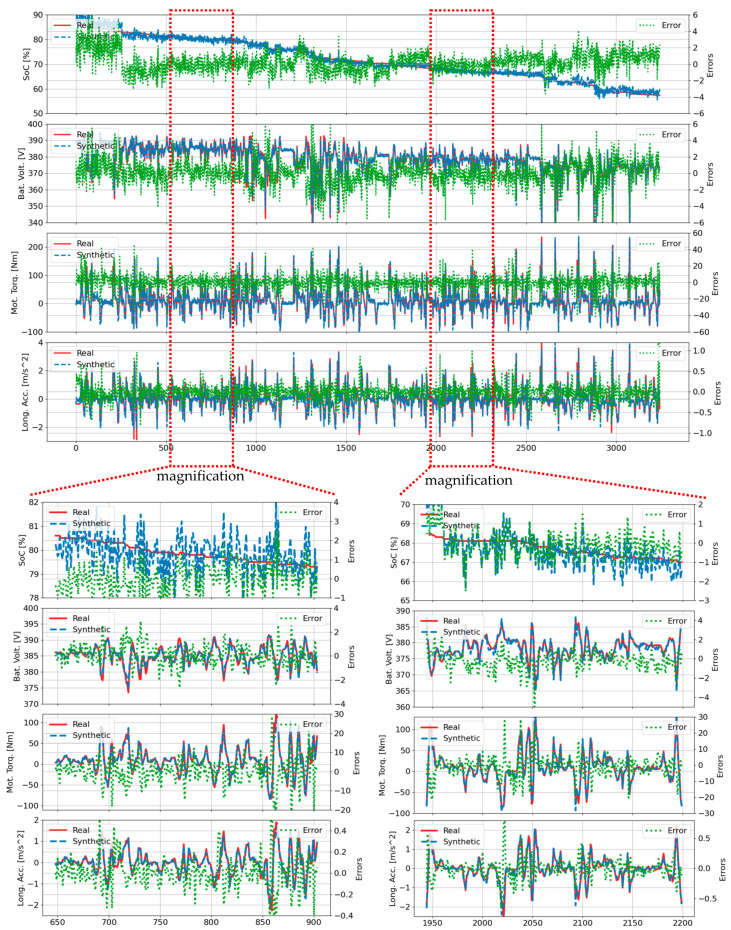
Comparison of real and synthetic data generated by TTS-GAN [41] for the B01 trip, trained exclusively on the B01 trip data.

**Figure 5:**
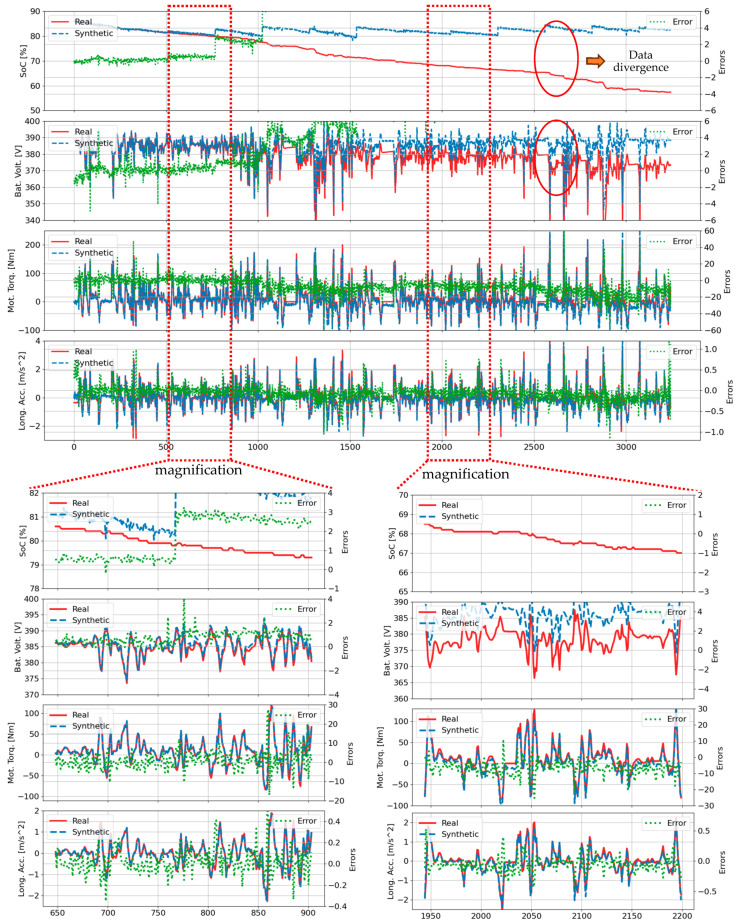
Comparison of real and synthetic data generated by TTS-GAN [41] for the B01 trip, trained using data from all trips.

Figure 4 and Figure 5 present comparative results generated by the transformer-based time-series generative adversarial network (TTS-GAN) model [41] for trip B01. Figure 4 showcases results trained exclusively on trip B01 data, while Figure 5 displays results trained on all 70 trips. In Figure 4, the synthetic data closely matches the real data for most features, with the SOC feature showing notable exceptions. The magnification plots in the lower left and right demonstrate that while the SOC feature exhibited larger deviations compared to other features, it still followed the general trend of the real SOC data. In Figure 5, the synthetic SOC values began to diverge from the ground truth data after time step 770. The battery voltage feature also showed divergence, though this began later at time step 1500 and exhibited notably smaller deviations compared to the SOC feature. The TTS-GAN model demonstrates the capability to synthesize multivariate realistic EV driving data for the B01 trip. However, while TTS-GAN models have successfully addressed simplified scenarios, these approaches often fail when applied to diverse real-world datasets. In LIB applications, voltage characteristics during charge and discharge cycles exhibit distinctive plateau regions that are crucial for understanding battery voltage and SOC estimation. Due to the plateau effect, it is challenging to accurately estimate the initial SOC level or battery voltage under various driving scenarios. By leveraging these multiple data streams, the TS-p2pGAN model can deliver accurate SOC estimations across varying driving conditions.

**Figure 6:**
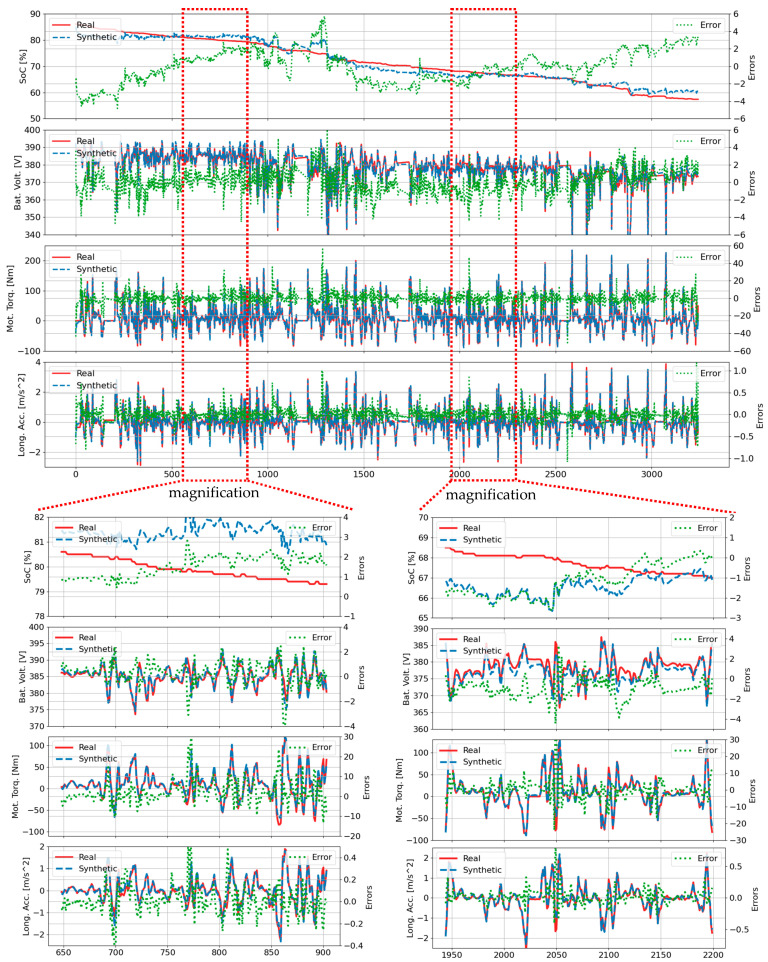
Comparison of real and synthetic data generated by Time-GAN [26] for the B01 trip.

Figure 6 presents comparative results generated by the TimeGAN model [26] for identical parameters. TimeGAN’s synthetic data exhibited notably more volatile behavior. Although it captured some fundamental parameter characteristics, the model struggled to accurately reproduce both local trends and broader temporal dynamics of the time-series data. The TS-p2pGAN model consistently achieved superior accuracy across all four parameters compared to TimeGAN.

Table 4 compares the performance metrics of TS-p2pGAN, TTS-GAN, and TimeGAN across 70 trips, revealing significant differences in model performance. Group B trips, labeled B01 to B36, correspond to trip numbers 1 to 36, while Group A trips, labeled A01 to A32, correspond to trip numbers 37 to 70. For trip 1, the data generated by TTS-GAN, trained exclusively on the B01 trip data, are shown in the parentheses. The RMSE, MAE, and DTW values were calculated according to Equations (5)–(7). TS-p2pGAN consistently demonstrated superior performance, with lower RMSE, MAE, and DTW values, indicating its effectiveness in modeling synthetic data. In contrast, TTS-GAN exhibited variable performance, performing poorly when trained on the full dataset and less consistently than TS-p2pGAN. Notably, for trip 1, when TTS-GAN was trained exclusively on B01 data (values in parentheses), its performance significantly deteriorated, underscoring the model’s difficulty in generalizing effectively when trained on broader datasets.

TimeGAN achieved RMSE values predominantly below 3% and MAE values under 1.5%. In contrast, TimeGAN faced substantial computational limitations, requiring individual training on smaller subsets of trips (trips 1–16) rather than the entire dataset, which resulted in significantly higher error rates, particularly evident in trips 4 and 9 where RMSE values exceeded 20%. While models can generate SOC waveforms for single-trip scenarios, TimeGAN encounters significant challenges when dealing with multitrip datasets featuring diverse driving conditions and drifting SOC values caused by varying initial SOC levels. These limitations result in higher error rates and reduced adaptability.

The comparison between Group B trips (B01 to B36, trip numbers 1 to 36) and Group A trips (A01 to A32, trip numbers 37 to 70) revealed more consistent model performance in Group B, while Group A trips showed greater variability, especially in complex scenarios. Overall, TS-p2pGAN outperformed the other models, demonstrating robustness across a diverse range of trips.

The performance disparity became most pronounced in complex scenarios with multiple start−stop conditions, as evidenced in trip B01 (Figure 3, Figure 4, Figure 5 and Figure 6) where TS-p2pGAN achieved an RMSE of 1.96%, MAE of 0.97%, and TDW of 1.19%, substantially outperforming TTS-GAN, which exhibited higher error rates of 3.87%, 2,62%, and 2.70%, respectively, and TimeGAN, with the respective error rates of 3.67%, 2.60%, and 2.84%. While both TTS-GAN and TimeGAN struggled with complex trip characteristics, TS-p2pGAN maintained remarkable consistency, keeping RMSE values below 2% even during challenging trip segments. The comprehensive metrics in Table 4 provide conclusive evidence of TS-p2pGAN’s superior capabilities, demonstrating consistently lower error rates across all evaluated trips and underscoring its effectiveness in handling complex, real-world EVs driving data scenarios.

**Figure 7:**
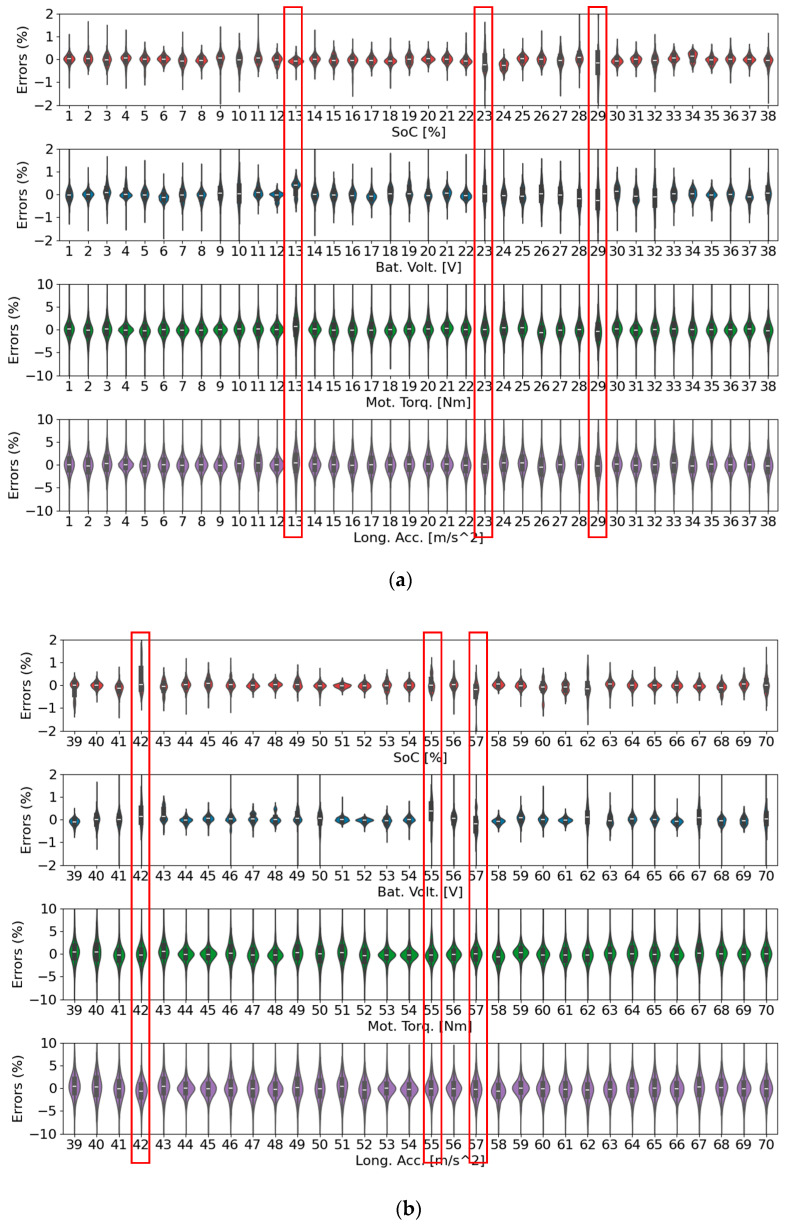
Violin plots show error distributions for four features across all trips: (**a**) Group B (trips B01 to B38); (**b**) group A (trips A01 to A32).

Error distribution analysis across 70 trips is visualized in Figure 7 through violin plots for four key features generated by TS-p2pGAN. The plot width indicates data density, with broader sections representing higher concentrations of data points. Error margins remained predominantly within ±1% for SOC and battery voltage, while motor torque and longitudinal acceleration showed wider variations up to ±5%. Most error distributions centered around zero, suggesting balanced positive and negative deviations. However, trips 13, 23, 29, 42, 44, and 57 (highlighted in red boxes) showed notable deviations from the expected bell-shaped distribution, indicating varying model accuracy across different driving scenarios.

**Figure 8:**
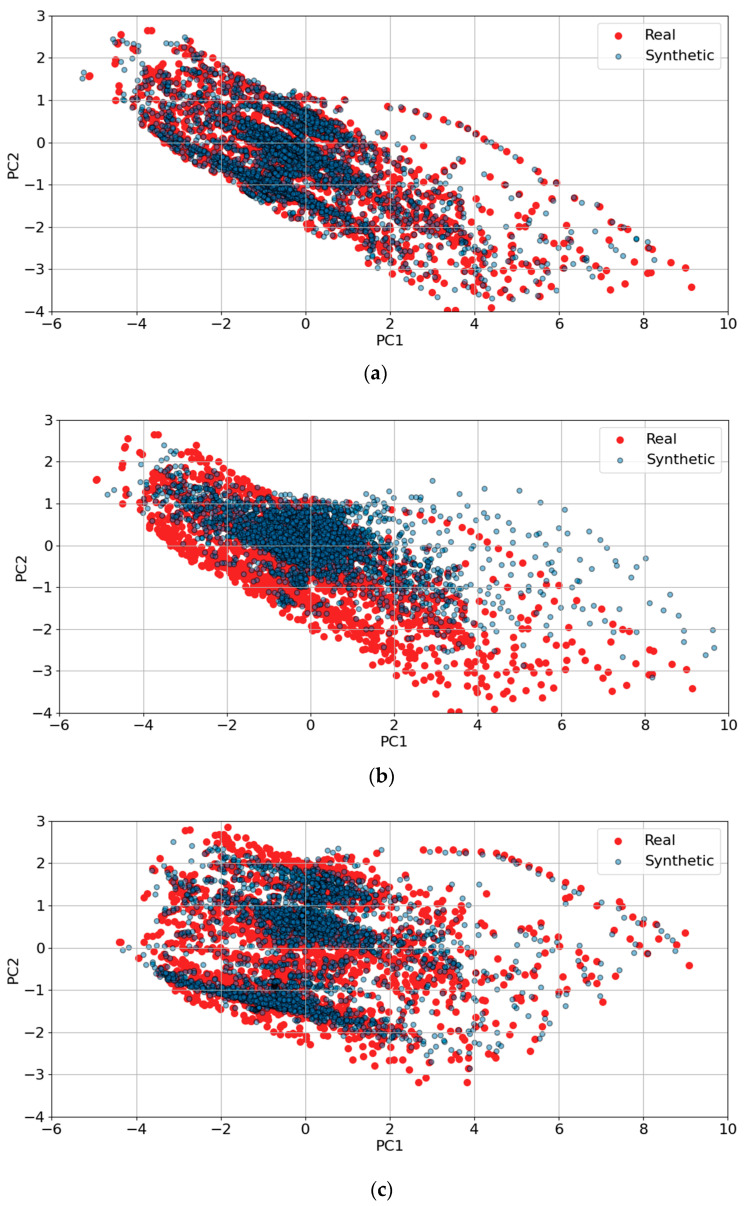
PCA visualizations of synthetic trajectory generation results for trip B01 using three different GAN architectures: (**a**) TS-p2pGAN; (**b**) TTS-GAN; (**c**) Time-GAN.

Figure 8 illustrates the effectiveness of different GAN architectures in replicating real-world driving trajectories for trip B01. The axes, labeled “PC1” (Principal Component 1) and “PC2” (Principal Component 2), represent the two principal components derived from PCA. PCA is a dimensionality reduction technique that projects high-dimensional data onto a lower-dimensional space while preserving as much variance as possible. Here, PC1 and PC2 capture the dominant patterns in the trajectory data, enabling a visual comparison of the real and synthetic data distributions.

The plots compare the synthetic trajectory results generated by TS-p2pGAN, TTS-GAN, and TimeGAN. In these visualizations, red points represent the real trajectory data, while blue points correspond to the synthetic data generated by the GANs. The degree of overlap between the red and blue distributions indicates how accurately the GAN replicates the characteristics of the real data.

Significantly, these results demonstrated distinct performance differences among the three models. In Figure 8a, TS-p2pGAN exhibited a high degree of alignment between the real and synthetic data, indicating its ability to faithfully capture complex trajectory patterns. In contrast, Figure 8b,c showed that TTS-GAN and TimeGAN exhibited less overlap, suggesting their limitations in accurately reproducing the real trajectory features. These findings underscore the superior performance of TS-p2pGAN in generating realistic synthetic trajectories, a crucial capability for applications requiring high-quality synthetic data, such as training and validating EV driving models.

**Figure 9:**
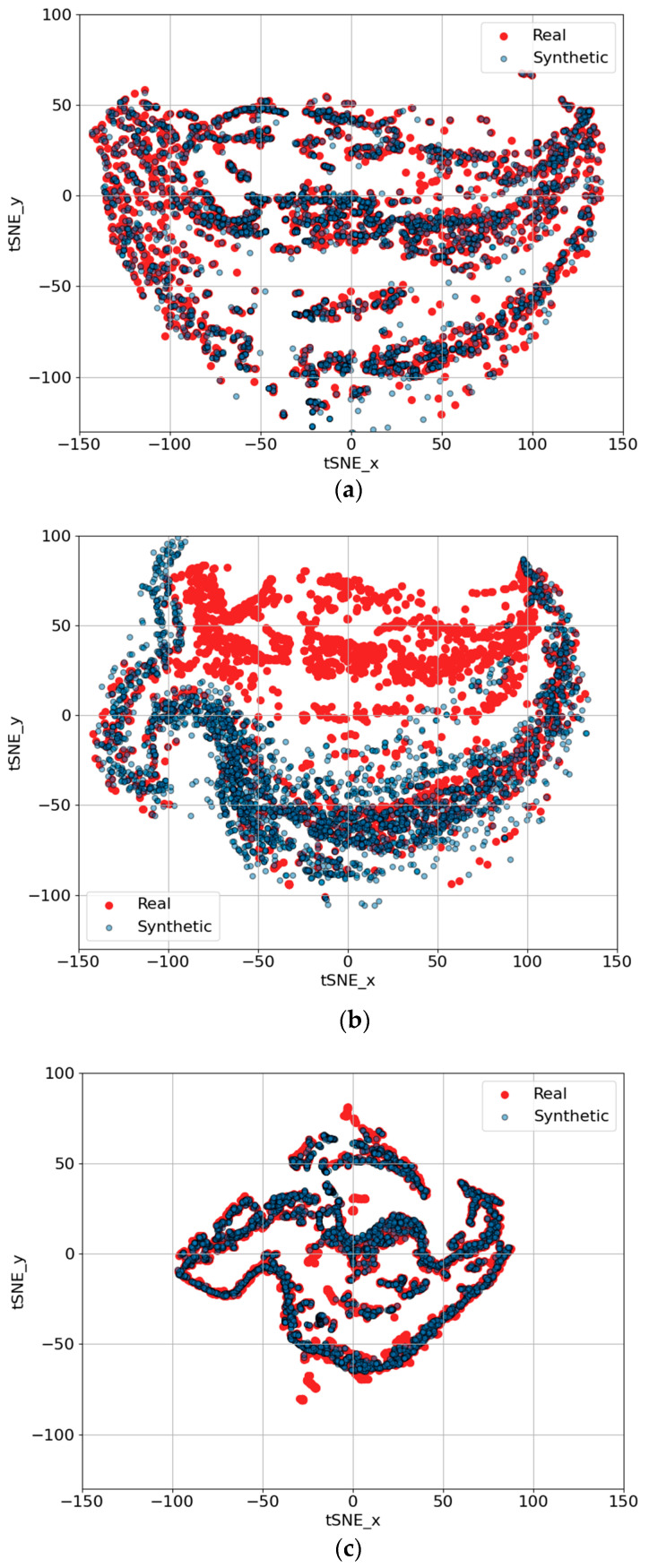
t-SNE visualizations of synthetic trajectory generation results for trip B01 using three different GAN architectures: (**a**) TS-p2pGAN; (**b**) TTS-GAN; (**c**) Time-GAN.

Figure 9 provides a deeper understanding of the performance of the three GAN architectures—TS-p2pGAN, TTS-GAN, and TimeGAN—in generating synthetic trajectories for trip B01, as visualized using t-SNE. The axes, labeled “tSNE_x” and “tSNE_y”, represent the two-dimensional projection of high-dimensional data, where t-SNE is used to preserve local and global structure, such as clusters or patterns in the data, in a lower-dimensional space for visual analysis.

In these visualizations, red points represent the real trajectory data, while blue points denote the synthetic data generated by the respective GANs. The degree of overlap between the real and synthetic distributions reflects how closely the generated data resemble the real data. Figure 9a shows that TS-p2pGAN achieved a high degree of alignment between the real and synthetic data, indicating its ability to accurately replicate the underlying trajectory patterns. Figure 9b, corresponding to TTS-GAN, demonstrated partial overlap with visible gaps and mismatches, suggesting weaker performance in capturing complex trajectory characteristics. Figure 9c, representing TimeGAN, exhibited a moderate alignment but with notable inconsistencies in some regions, further highlighting its limitations.

These results further validated TS-p2pGAN’s superior performance. By maintaining closer proximity to the real data in the t-SNE space, TS-p2pGAN demonstrated its effectiveness in capturing intricate temporal and spatial dependencies within the driving trajectories. These findings underscore the practical utility of TS-p2pGAN in generating realistic synthetic trajectories for applications such as EV driving data modeling and simulation.

**Figure 10:**
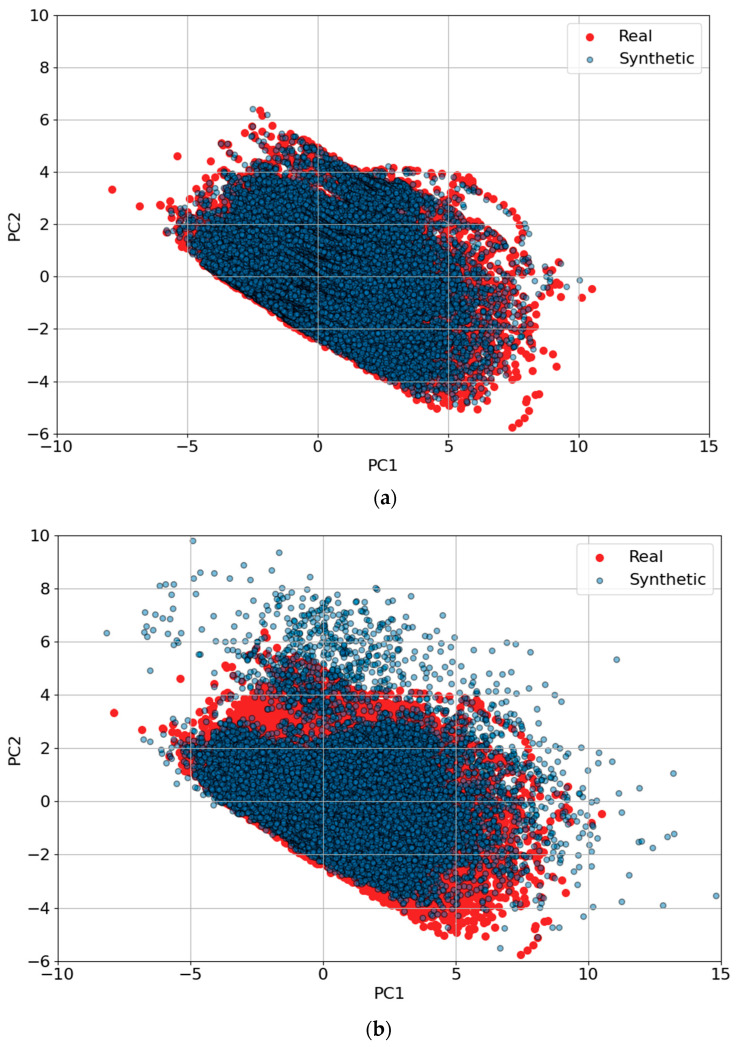
PCA visualizations comparing synthetic trajectory generation results for all trips using GAN architectures: (**a**) TS-p2pGAN; (**b**) TTS-GAN.

**Figure 11:**
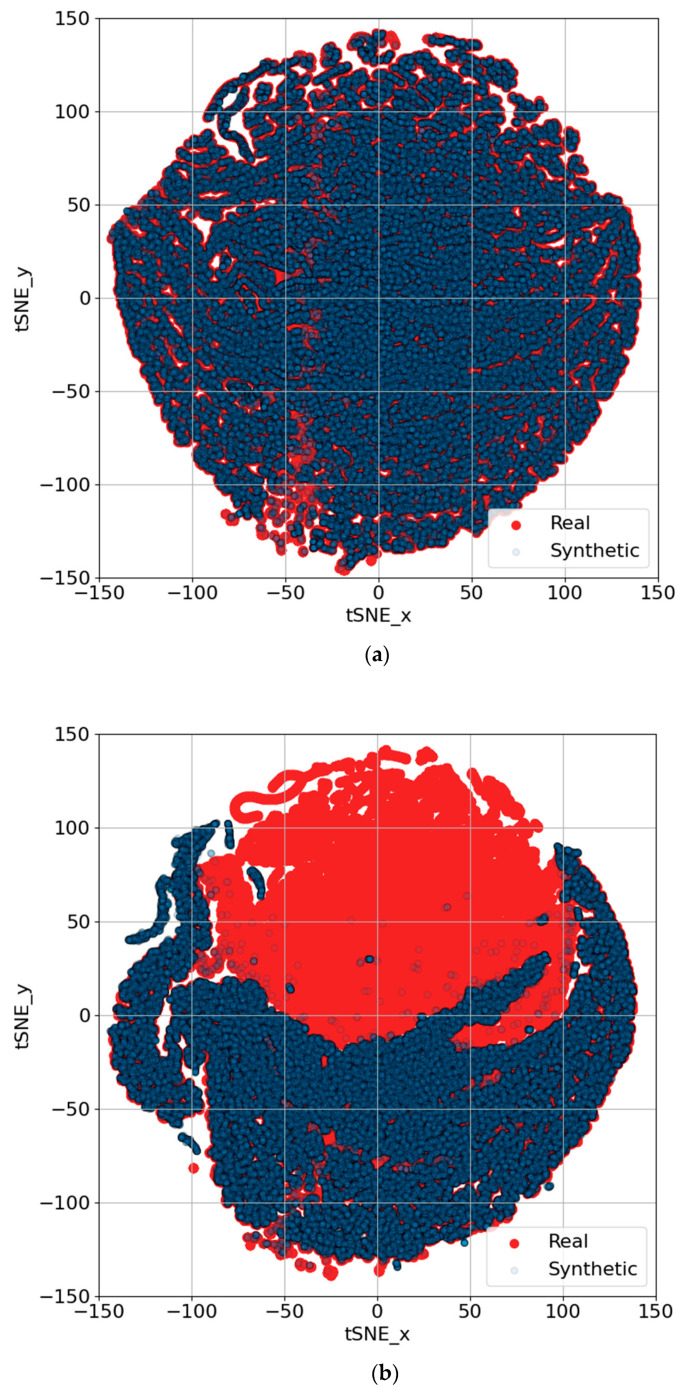
t-SNE visualizations comparing synthetic trajectory generation results for all trips using GAN architectures: (**a**) TS-p2pGAN; (**b**) TTS-GAN.

Figure 10 and Figure 11 present PCA and t-SNE plots, respectively, extending the analysis to encompass synthetic data generation across all trips using both TS-p2pGAN and TTS-GAN. Figure 10a demonstrates that the synthetic data generated by TS-p2pGAN (blue) exhibited significant overlap with the real data (red) in the PCA space. This indicated that the TS-p2pGAN model effectively captured the overall distribution and variance of the original dataset. In contrast, Figure 10b shows that while TTS-GAN successfully captured overall trends, it produced more dispersed distributions, particularly around core areas, suggesting less precise replication of the original data patterns. The presence of real data points beyond the synthetic distribution in both figures suggests that the models may not fully replicate more complex or rare trajectory patterns. The tight clustering of synthetic data around the core of the real data distribution reflects the models’ ability to learn the general structure but also highlights potential limitations in generating diverse outliers and behaviors.

Figure 11 provides t-SNE visualizations comparing the effectiveness of two GAN architectures, TS-p2pGAN (Figure 11a) and TTS-GAN (Figure 11b), in generating synthetic trajectories. In Figure 11a, TS-p2pGAN demonstrates significant overlap with the real data, with only slight scattering at the distribution’s edges, indicating minor challenges in capturing extreme cases. In contrast, Figure 11b reveals that the synthetic data generated by TTS-GAN formed two distinct clusters, failing to align with the real data distribution. This suggests that TS-p2pGAN produced synthetic data more closely aligned with the real data, while TTS-GAN struggled to capture the full variability, resulting in noticeable clustering. These findings underscore the differing abilities of the two architectures in synthesizing realistic multivariate time-series data.

Overall, these results demonstrate that the GAN models effectively capture the general behavior of trajectory data but require further refinement to better reproduce intricate and uncommon patterns. This performance is promising for synthetic data generation but underscores the need for improvements in high-precision tasks. These insights are crucial for applications that depend on realistic synthetic data, such as simulation-based testing.

## 4. Conclusions

The challenge of limited access to real-world data significantly impacts machine learning applications in EV and power battery dynamics analysis, as traditional time-series data augmentation methods often struggle to maintain essential signal characteristics while expanding datasets. To address this challenge, TS-p2pGAN, a novel model designed for generating variable-length synthetic time-series data while preserving original signal properties, is introduced. The model’s architecture uniquely incorporates a transformation net generator for point-to-point translation, utilizing gradient flow from multiple discriminators to a single generator across various scales to effectively capture complex EV parameter influences, including SOC and motor output torque.

Validation, conducted using an open dataset of 70 EV driving trips with comprehensive battery condition data, demonstrated TS-p2pGAN’s superior performance compared to TTS-GAN and TimeGAN in generating realistic and accurate time-series data. The model particularly excelled in preserving temporal dynamics, crucial for maintaining inter-variable relationships across time sequences. Quantitative analysis revealed impressive results, with RMSE values consistently below 3% and MAE values under 1.5% across all trips. Qualitative assessments through t-SNE and PCA visualizations further confirmed the high fidelity of generated data, while discriminative and predictive capability tests highlighted TS-p2pGAN’s advantages over TimeGAN in time-series generation.

The practical implications of TS-p2pGAN extend beyond data generation, offering significant potential for enhancing SOC and motor torque estimation, ultimately contributing to EV energy consumption optimization. The model’s ability to effectively leverage both spatial and temporal features surpasses traditional methods in learning complex time-series patterns while maintaining data integrity.

Despite these achievements, TS-p2pGAN’s reliance on paired datasets presents a notable limitation in diverse real-world environments where such data are often scarce. Future research could enhance the model’s versatility by exploring integration with unpaired learning approaches, particularly through CycleGAN architectures augmented with physics-informed constraints. While this limitation exists, the model demonstrates remarkable robustness through its ability to generate synthetic parameters that maintain consistency with both physical constraints and vehicle dynamics across various driving conditions. Ultimately, TS-p2pGAN marks a breakthrough in synthetic time-series generation for electric vehicle applications, delivering a framework that successfully balances high fidelity, practical utility, and real-world applicability.

Future work could explore the application of this data augmentation framework across diverse domains, including digital energy management systems for LIBs, autonomous vehicle comfort systems, and broader EV technologies. Real-time implementation and validation of TS-p2pGAN in diverse on-road scenarios are crucial to evaluate its performance under dynamic and variable conditions. Collaborating with industries, such as automotive manufacturers, and integrating the framework into existing control systems can significantly enhance its practical utility. The framework’s ability to generate synthetic parameters that adhere to physical constraints and maintain consistency with underlying system dynamics across different conditions demonstrates its robustness and potential for wide-ranging applications. Furthermore, the framework can be employed to accurately estimate SOC for LIBs’ battery management systems (BMSs), improving their reliability and efficiency. This offers significant contributions to fields requiring high-fidelity synthetic time-series data, particularly in enhancing the performance of LIBs and BMS technologies.

## Figures and Tables

**Table 1:** The key parameters of the BMW i3 (60 Ah) EV [36].

Parameter	Specification
Power	170 horsepower (125 kW)
Torque	184 lb-ft (250 Nm)
Acceleration (0–60 mph)	7.3 s
Top speed	93 mph (150 km/h)
Battery type	Lithium-ion
Battery capacity	60 Ah, 33 kWh (usable)
Real-world range	Approximately 115 km (71 miles)
Length	3999 mm (157.4 inches)
Width	1775 mm (69.9 inches)
Height	1578 mm (62.1 inches)
Wheelbase	2570 mm (101.2 inches)
Curb weight	1195 kg (2635 lbs)
AC charging capacity	11 kW (optional 7.4 kW)
DC fast charging	50 kW (optional 11 kW)
Charging time (0–100%)	Approximately 3 h (using a 7.4 kW charger)

**Table 2:** Hyperparameters of the multiscale generator model.

Name	Layer	(k, s, p)	f	d	Module
Input			10	256	Front-end $\left(G_{F}\right)$ Downsampling operations
Reflect_1	Pad1d	(-, -, 3)	10	262	
Conv_1	Conv1d_N+R_	(7, 1, 0)	64	256	
Conv_2	Conv1d_N+R_	(3, 2, 1)	128	128	
Conv_3	Conv1d_N+R_	(3, 2, 1)	256	64	
Conv_4	Conv1d_N+R_	(3, 2, 1)	512	32	
Conv_5	Conv1d_N+R_	(3, 2, 1)	1024	16	
ResnetBlock_1	Pad1d Conv1d_N+R_ Pad1d Conv1d_N+R_	(-, -, 1) (3, 1, 1) (-, -, 1) (3, 1, 1)	1024 1024 1024 1024	16 18 16 16	Residual blocks ($G_{R}$)
ResnetBlock_2	Pad1d Conv1d_N+R_ Pad1d Conv1d_N+R_	(-, -, 1) (3, 1, 1) (-, -, 1) (3, 1, 1)	1024 1024 1024 1024	16 18 16 16	
ResnetBlock_3	Pad1d Conv1d_N+R_ Pad1d Conv1d_N+R_	(-, -, 1) (3, 1, 1) (-, -, 1) (3, 1, 1)	1024 1024 1024 1024	16 18 16 16	
ResnetBlock_4	Pad1d Conv1d_N+R_ Pad1d Conv1d_N+R_	(-, -, 1) (3, 1, 1) (-, -, 1) (3, 1, 1)	1024 1024 1024 1024	16 18 16 16	
ResnetBlock_5	Pad1d Conv1d_N+R_ Pad1d Conv1d_N+R_	(-, -, 1) (3, 1, 1) (-, -, 1) (3, 1, 1)	1024 1024 1024 1024	16 18 16 16	
ResnetBlock_6	Pad1d Conv1d_N+R_ Pad1d Conv1d_N+R_	(-, -, 1) (3, 1, 1) (-, -, 1) (3, 1, 1)	1024 1024 1024 1024	16 18 16 16	
ResnetBlock_7	Pad1d Conv1d_N+R_ Pad1d Conv1d_N+R_	(-, -, 1) (3, 1, 1) (-, -, 1) (3, 1, 1)	1024 1024 1024 1024	16 18 16 16	
ResnetBlock_8	Pad1d Conv1d_N+R_ Pad1d Conv1d_N+R_	(-, -, 1) (3, 1, 1) (-, -, 1) (3, 1, 1)	1024 1024 1024 1024	16 18 16 16	
ResnetBlock_9	Pad1d Conv1d_N+R_ Pad1d Conv1d_N+R_	(-, -, 1) (3, 1, 1) (-, -, 1) (3, 1, 1)	1024 1024 1024 1024	16 18 16 16	
ConvTran_1	ConvTranspose1d_N+R_	(3, 2, 1)	512	32	Back-end {$G_{B}$). Upsampling operations
ConvTran_2	ConvTranspose1d_N+R_	(3, 2, 1)	256	64	
ConvTran_3	ConvTranspose1d_N+R_	(3, 2, 1)	128	128	
ConvTran_4	ConvTranspose1d_N+R_	(3, 2, 1)	64	256	
Reflect_2	Pad1D	(-, -, 3)	64	262	
Conv_6	Conv1D	(7, 1, 0)	4	256	
Tanh_1	Tanh		4	256	

Note: k, kernel; s, stride; p, padding; f, feature; d, dimension; Conv1d_N+R_, Conv1d + BatchNorm1d + ReLU; ConvTranspose1d_N+R_, ConvTranspose1d + BatchNorm1d + ReLU.

**Table 3:** Hyperparameters of the multiscale discriminator model.

Name	Layer	(k, s, p)	f	d	Scale
Input			14	256	scale 1
Conv_1	Conv1d_R_	(4, 2, 2)	64	129	
Conv_2	Conv1d_N+R_	(4, 2, 2)	128	65	
Conv_3	Conv1d_N+R_	(4, 2, 2)	256	33	
Conv_4	Conv1d_N+R_	(4, 1, 2)	512	34	
Conv_5	Conv1d	(4, 1, 2)	1	35	
Sigmoid_1	Sigmoid		1	35	
Input			14	256	scale 2
Pool1D_1	AvgPool1D	(3, 2, 1)	14	128	
Conv_6	Conv1d_R_	(4, 2, 2)	64	65	
Conv_7	Conv1d_N+R_	(4, 2, 2)	128	33	
Conv_8	Conv1d_N+R_	(4, 2, 2)	256	17	
Conv_9	Conv1d_N+R_	(4, 1, 2)	512	18	
Conv_10	Conv1d	(4, 1, 2)	1	19	
Sigmoid_2	Sigmoid		1	35	

Note: k, kernel; s, stride; p, padding; f, feature; d, dimension; Conv1dR, Conv1d + Leaky ReLU; Conv1dN + R, Conv1d + BatchNorm1d + Leaky ReLU.

**Table 4:** RMSE, MAE, and DTW values for all trips.

Trip No	TS-p2pGAN			TTS-GAN			TimeGAN			Trip No	TS-p2pGAN			TTS-GAN		
	RMSE (%)	MAE (%)	DTW (%)	RMSE (%)	MAE (%)	DTW (%)	RMSE (%)	MAE (%)	DTW (%)		RMSE (%)	RMAE (%)	DTW (%)	RMSE (%)	MAE (%)	DTW (%)
1	1.96	0.97	1.19	18.57 (3.87)	11.61 (2.62)	13.20 (2.70)	3.67	2.60	2.84	36	1.75	0.86	1.00	11.70	8.47	8.50
2	2.02	1.01	1.12	13.25	9.94	10.68	3.94	2.81	2.93	37	1.89	0.93	1.13	35.53	24.78	31.31
3	1.91	1.02	1.21	31.09	22.51	27.46	5,35	3.83	3.45	38	2.30	1.13	1.36	3.62	2.21	2.56
4	1.79	0.77	0.84	46.13	31.66	30.31	31.48	18.56	17.2	39	2.17	1.18	1.40	42.53	33.33	25.59
5	1.91	0.92	1.09	24.69	17.64	21.47	7.45	5.34	5.50	40	2.45	1.33	1.67	7.54	5.36	6.39
6	1.62	0.84	1.03	12.75	9.33	10.69	7.94	5.80	6.26	41	2.40	1.19	1.48	16.69	12.24	15.32
7	1.56	0.84	1.01	31.13	20.87	24.75	4.79	3.44	3.33	42	2.71	1.26	1.40	27.03	18.74	26.44
8	1.51	0.78	0.92	35.81	23.64	28.84	5.81	4.10	3.71	43	2.33	1.22	1.48	14.79	9.07	9.58
9	1.73	0.85	0.95	49.62	32.90	32.61	25.78	15.63	9.57	44	1.47	0.77	0.90	50.96	33.71	39.99
10	2.03	1.06	1.26	32.81	19.02	18.78	6.89	5.18	4.52	45	1.58	0.79	0.93	6.56	4.31	5.19
11	2.08	1.01	1.15	62.69	43.97	59.21	7.94	6.12	5.60	46	2.41	1.20	1.39	16.99	11.57	16.62
12	1.26	0.66	0.72	31.30	25.48	21.52	11.27	8.18	5.94	47	2.28	1.08	1.30	24.72	16.80	22.56
13	2.69	1.46	1.56	37.27	27.90	36.33	6.36	3.89	3.06	48	1.72	0.85	1.00	8.40	5.53	6.30
14	1.48	0.80	0.91	37.71	22.43	21.33	6.96	4.95	4.46	49	2.58	1.24	1.45	19.53	13.56	17.77
15	1.83	0.96	1.17	9.80	6.53	6.95	7.30	5.01	4.67	50	3.29	1.46	1.75	2.58	1.46	1.67
16	1.92	1.02	1.21	28.90	21.91	25.54	6.76	4.98	4.80	51	2.19	1.15	1.39	5.22	3.98	4.81
17	2.11	1.07	1.28	49.46	35.62	44.59				52	2.21	1.09	1.30	22.12	14.56	18.67
18	1.66	0.87	1.01	12.46	8.00	9.99				53	1.58	0.79	0.94	38.20	25.29	33.19
19	1.80	0.92	1.09	8.55	5.85	6.19				54	1.45	0.73	0.87	5.08	3.72	3.96
20	1.91	0.96	1.11	21.95	15.79	19.62				55	2.31	1.14	1.27	14.24	9.94	12.81
21	1.56	0.80	0.97	44.54	29.58	39.98				56	1.48	0.84	0.99	4.89	3.07	3.52
22	2.05	0.97	1.20	10.73	7.62	8.64				57	2.54	1.24	1.45	16.64	11.41	15.79
23	2.65	1.31	1.52	30.09	21.17	25.40				58	1.75	0.94	1.15	44.50	29.56	39.91
24	2.36	1.25	1.42	41.96	29.39	39.62				59	1.55	0.79	0.97	5.83	3.35	4.01
25	2.23	1.09	1.26	58.61	40.63	54.61				60	2.05	1.00	1.19	3.29	2.26	2.50
26	2.75	1.30	1.51	74.71	51.57	69.51				61	2.05	1.03	1.27	9.19	7.27	7.98
27	2.04	1.11	1.30	49.42	32.70	44.03				62	2.78	1.27	1.43	20.50	14.73	18.37
28	2.21	1.23	1.40	70.08	46.91	64.95				63	1.87	0.99	1.21	4.19	2.67	3.24
29	2.90	1.67	1.77	82.92	56.46	76.35				64	2.04	1.06	1.35	16.53	11.77	15.13
30	2.25	1.02	1.22	8.66	5.65	6.45				65	2.52	1.16	1.45	11.00	7.59	10.04
31	1.86	0.87	1.06	25.35	17.74	21.54				66	1.63	0.87	1.09	21.60	14.68	20.88
32	2.70	1.30	1.52	48.52	32.72	44.29				67	2.74	1.37	1.69	3.35	2.23	2.52
33	2.45	1.24	1.60	11.11	8.21	10.31				68	2.16	1.10	1.35	11.02	7.69	9.05
34	2.32	1.12	1.32	12.58	8.33	12.32				69	1.88	0.96	1.22	19.09	11.41	12.81
35	1.95	0.93	1.08	10.59	7.37	8.39				70	2.37	1.13	1.34	11.70	8.47	8.50

Note: Data within parentheses were generated by TTS-GAN, trained exclusively on the B01 trip data. Group B trips, labeled B01 to B36, correspond to trip numbers 1 to 36, while Group A trips, labeled A01 to A32, correspond to trip numbers 37 to 70.

## Data Availability

Data are available upon request.
